# Myeloid HIF1α has only a minor impact on atherosclerosis development

**DOI:** 10.1530/VB-25-0011

**Published:** 2026-01-24

**Authors:** Nathalie Dehne, Katrin Schröder

**Affiliations:** ^1^ Fakultät Für Gesundheitswesen, Universität Potsdam, Campus Golm, Potsdam Golm Germany; ^2^ Institute for Cardiovascular Physiology, Goethe-University Frankfurt, Frankfurt am Main, Germany

**Keywords:** HIF, macrophages, atherosclerosis

## Abstract

Atherosclerosis is a progressive inflammatory disease, of which initiation and progression are potentially mediated by myeloid cells. An imbalance of oxygen supply and, therefore, hypoxic situations in the arterial wall have been hypothesized to be a major driver of development and progression of atherosclerosis. Herein, we analyze the significance of hypoxia-inducible factor (HIF) in myeloid cells in atherosclerosis. Myeloid-specific Hif1α and Hif2α knockout mice were crossed into the ApoE^−/−^ background, and angiotensin II (AngII) infusion was performed to induce accelerated plaque formation. Myeloid Hif1α, but not Hif2α, limited the increase in heart weight after 7 days of AngII infusion, indicating a transient protective effect restricted to early phases of AngII-induced remodeling. With prolonged treatment (4 weeks), these differences were lost, suggesting a protective role for myeloid HIF-1α only in early hypertension-induced cardiac hypertrophy. Macrophages of aged mice (12 months old) showed decreased expression of Hif1α and Hif2α, which did not yield overt differences in classical/alternative polarization markers. Nevertheless, aged ApoE^−/−^ mice with macrophage-specific Hif1α knockout had a higher body weight and developed more aortic plaques compared to wild-type littermates. These observations suggest that activation of Hif1α in macrophages may be protective for plaque formation under chronic hyperlipidemic conditions. Supporting this, a reanalysis of single-cell RNA-sequencing data from human atherosclerotic and normal vessel wall specimens shows that HIF target gene expression is elevated in anti-inflammatory macrophage subsets along pseudotime trajectories. This association suggests that macrophage HIF1α activity may contribute to reparative or stabilizing responses during plaque progression.

## Introduction

Atherosclerosis-associated cardiovascular diseases are the leading cause of death in the western world. The disease can start with an activation of endothelial cells (ECs), for example upon an injury, in response to inflammation, or in response to pro-atherogenic lipids. This is followed by an increased proliferation of smooth muscle cells (SMCs) and invasion of monocytes that accumulate modified lipids and thus become foam cells (1, 2). In advanced lesions, the accumulation of extracellular matrix and lipid particles create a microenvironment that leads to cell death, reduced efferocytosis, and infiltration of additional monocytes (3). In general, atherosclerotic lesion size correlates with the number of circulating monocytes and inhibition of monocyte recruitment abolishes atherosclerotic development at least in hypercholesterolemic mice, emphasizing the importance of these cells for disease progression (4).

One hypothesis of plaque formation and progression assumes an imbalance of oxygen supply in the arterial wall as causal for the development of atherosclerosis (5). Areas with low oxygen were demonstrated in atherosclerotic plaques (6, 7). This is accompanied by the accumulation of hypoxia-inducible factor (HIF) in the perivascular tissue, especially in macrophage-rich areas of the atheromatous core. In the mammalian system, three HIF isoforms are described, which differ in their α-subunit but which all dimerize with the β-subunit ARNT (aryl hydrocarbon receptor nuclear translocator). HIF1α is ubiquitously expressed and the best studied isoform. Expression of HIF2α is restricted to certain cell types, such as ECs and macrophages, while HIF3α is considered a negative regulator or modulator of the system (8). The α-subunit is the oxygen-dependent regulated part of the HIF transcription factors (9). In the presence of sufficient levels of oxygen, the α-subunit is hydroxylated by prolyl hydroxylases (PHDs) and subsequently subjected to proteasomal degradation. Prolyl hydroxylases initiate cellular hypoxic responses, influencing macrophage function in plaque hypoxia. However, each isoform fulfills different tasks in macrophages: myeloid PHD2cko and PHD3ko enhanced atherosclerotic plaque growth and macrophage apoptosis, while PHD2cko macrophages further activated collagen secretion by fibroblasts (10). In addition, myeloid PHD2 conditional knockout improves intraplaque angiogenesis and vascular remodeling (11). At low oxygen levels, PHDs are inhibited and the α-subunit accumulates and translocates to the nucleus, where it dimerizes with the β-subunit. HIF1 and HIF2 act as transcription factors and induce expression programs that increase oxygen supply and support survival in hypoxic areas (12). Hypoxia within expanding plaques promotes intraplaque angiogenesis and vasa vasorum neovascularization, processes that correlate with macrophage-rich inflammatory regions and Hif1α abundance in human lesions (13). Neovessel formation can exacerbate lipid influx and intraplaque hemorrhage, further shaping macrophage phenotypes under hypoxic stress. In addition, HIF target genes have been implicated in the promotion of atherosclerosis by increasing SMC proliferation and migration, neovessel formation, alterations in cellular metabolism, and calcification of blood vessels (5). Consistently, local application of siRNA to reduce Hif1α expression or cell type-specific knockout in ECs or SMCs significantly decreased plaque size and SMC and macrophage content (14).

By increasing lipid synthesis and lipid droplet formation and by reducing cholesterol efflux through the ATP-binding cassette transporter (ABCA-1) in macrophages, hypoxia may contribute to atherosclerosis progression (5). However, conflicting results have been published concerning the role of macrophage Hif1α in this process. Knockout of Hif1α in myeloid cells in mice with a low-density lipoprotein receptor (LDLR)-deficient background has been shown to be protective (7) and detrimental (6) for atherosclerosis development. This discrepancy may be explained by the type of diet applied: Chaudharir *et al.* (7) used an aggressive 15% fat and 1.25% cholesterol diet for 8 weeks, whereas Aarup *et al.* (6) used a milder 0.3% cholesterol and 4.3% fat diet for 16 weeks. On this basis, we hypothesized that the different inflammatory activity, induced by the chows, impacted the study’s results.

On this basis, we utilized two models of atherosclerosis development that are diet independent. First, to study accelerated atherosclerosis, we administered angiotensin II (AngII) to myeloid-specific Hif1α and Hif2α knockout mice crossed into the ApoE-deficient background to induce vessel inflammation and plaque formation over a short time (7 days) and a prolonged time (4 weeks). Second, to analyze age-dependent formation of atherosclerotic lesions, mice were fed a regular chow diet for 12 months.

## Materials and methods

### Mice

Hif1α^flox/flox^/lysozyme M Cre (LysMCre) mice (Hif1α^m−/−^) were generated and kindly provided by Prof RS Johnson (Department of Physiology, Development and Neuroscience, University of Cambridge, UK). Hif2α^flox/flox^ mice were obtained from Prof MC Simon (Department of Cancer Biology, University of Pennsylvania School of Medicine, Philadelphia, PA, USA) and crossed with LysMCre mice to obtain myeloid-specific Hif-2α knockout mice (Hif2α^m−/−^). Flox-positive, Cre recombinase-negative mice served as wild-type controls (denoted as wt mice). ApoE^−/−^ mice were purchased from Charles River (Germany) and crossed into the myeloid Hif1α and Hif2α lines. Experimental groups are composed of littermates either lacking or expressing Cre recombinase (knockout or wild-type). Animals (*n* ≥ 7 per group) were treated for up to 4 weeks with AngII (1.44 mg/kg/day) delivered using a mini-osmotic pump (Alzet, USA). A lethal dose of isoflurane was used as a method of sacrifice, no anesthesia and/or analgesia was applied, and no obvious suffering of the mice was observed. Animal experiments (F28/28) were performed with the approval of the Ethics Committee of the Goethe University’s medical faculty.

### Histology

Organs were harvested from ApoE^−/−^/wt, Hif1α^m−/−^, and Hif2α^m−/−^ mice and immediately fixed in 4% paraformaldehyde. Samples were dehydrated, embedded in paraffin, and sectioned. Antigens were retrieved by boiling of sections for 12 min in TRS buffer (DAKO, Hamburg, Germany). Endogenous avidin and biotin were blocked using a biotin-blocking system (DAKO). Primary anti-mouse F4/80 (eBioscience, Frankfurt, Germany) antibody was incubated at 4°C overnight. Sections were stained using a catalyzed signal amplification (CSA) kit (DAKO, USA) and Mayer’s hemalum (Sigma, Germany). Sections were analyzed using a Zeiss Axioskop 40 system and the AxioVision software (Carl Zeiss Microscopy, Germany).

Plaques were defined by white spots resulting from intimal thickening with foam cell accumulation. Manual counting at multiple anatomical sites within each aorta was chosen as the primary method because atherosclerotic lesions occur at several discrete locations in this model and accurate plaque number reflects the onset and progression of atherosclerotic burden. Aortic mass per length (mg/mm) also serves as a measure of total plaque burden.

### Macrophages isolation and culture

Primary mouse macrophages were isolated from spleen. Spleens were removed, and single-cell suspensions were prepared using PBS and the Medicon/MediMachine System (BD Bioscience, Germany) as described by the manufacturer. CD11b^+^ splenocytes were isolated by MACS separation using MACS LS columns (Miltenyi Biotec, Germany) and CD11b^+^ MicroBeads (Miltenyi Biotec) according to the manufacturer’s instructions. For differentiation, CD11b^+^ cells were cultured in RPMI 1640 supplemented with 10% FCS, 100 μg/mL streptomycin, 100 U/mL penicillin (all from PAA Laboratories, Germany), and 25 ng/mL M-CSF (PeproTech, Germany) for 5 days. Cells were cultured in a humidified atmosphere at approximately 80% of cell density.

### mRNA isolation and qPCR

Total RNA was isolated using PeqGold RNA Pure kit as described by the manufacturer (PeqLab Biotechnology, Germany) and stored at −80°C. Concentration and quality of the RNA were determined using the NanoDrop ND-100 spectrometer (PeqLab). Reverse transcription was performed with 1 μg RNA using the Maxima first-strand cDNA synthesis kit for qPCR (Thermo Scientific, Germany) and stored at −20°C. Quantitative real-time PCR (qPCR) was performed with iQ SYBR green supermix according to the manufacturer’s instructions (BioRad, Germany) on a CFX connect qPCR system (BioRad). Data were analyzed using the BioRad software, and tenfold dilutions of purified PCR products served as standards. The primers used are given in Table 1.

**Table 1:** Primer sequences used for quantitative real-time PCR. Primers are designed for 60°C annealing temperature. The product size varies from 150 to 508 bp.

Gene	Accession no.	Sequence
*TBP*	NM_013684	ctg acc act gca ccg ttg cca
		gac tgc agc aaa tcg ctt ggg a
*Fizz1* (*Retnlg*)	NM_181596	ccc ttc tca tct gca tct cc
		cag tag cag tca tcc cag ca
*iNOS* (*Nos2*)	NM_001313922	acc cta aga gtc aca aaa tgg c
		ttg atc ctc aca tac tgt gga cg
*ADM*	NM_009627	cgc agt tcc gaa aga agt gg
		cca gtt gtg ttc tgc tcg tcc
*HIF-1α*	NM_001313919	gaa atg gcc cag tga gaa aa
		agt cta gag atg cag caa gat ctc ggc
*HIF-2α* (*Epas1*)	NM_010137	tga gtt ggc tca tga gtt gc
		ttg ctg atg ttt tcc gac ag
*PHD2* (*Egln1*)	NM_053207	agc cat ggt tgc ttg tta cc
		ttg ggt tca atg tca gca aa

### Single-cell RNA-Seq reanalysis of human specimen

To investigate the expression dynamics of HIF target genes in macrophages within human atherosclerotic plaques, we reanalyzed publicly available single-cell RNA sequencing data from GSE159677 (15). This dataset includes carotid endarterectomy tissue from three patients providing both paired proximal adjacent (PA) and atherosclerotic core (AC) regions.

#### Data processing

Raw count matrices were downloaded from https://www.ncbi.nlm.nih.gov/geo/query/acc.cgi?acc=GSE159677 and processed using the Seurat (v5.0.1) R package as documented in a Git repository (https://github.com/KSchroeder-gh/Hif-in-macrophages). Data normalization, variable feature identification (FindVariableFeatures, method = ‘vst’), scaling, and dimensionality reduction (PCA and UMAP) were performed following standard workflows.

#### Cell type annotation and marker analysis

Clusters were identified using Seurat’s graph-based clustering (FindClusters) and annotated based on canonical marker genes. Macrophage clusters were used for further analysis.

#### Pseudotime analysis

To investigate potential differentiation trajectories, macrophage clusters were extracted and converted to a SingleCellExperiment object. The root of the trajectory was anchored in monocyte clusters.

#### HIF-score analysis

A curated list of experimentally validated HIF target genes was used to evaluate HIF protein activity at the single-cell level. Per-cell relative HIF-scores were computed using AddModuleScore to account for background gene expression distribution. Scores were visualized in UMAP-based pseudotime projections separately across PA and AC clusters.

### Statistical analysis

Experiments were repeated as indicated in the figures. Data are expressed as mean ± SEM. Statistically significant differences were calculated after analysis of variance (ANOVA) and Bonferroni’s test. Individual *P* values are given in the figure legends. No correction for multiple comparisons was applied.

## Results

### HIF-1α in macrophages is protective in the onset of AngII-induced cardiovascular remodeling

**Figure 1:**
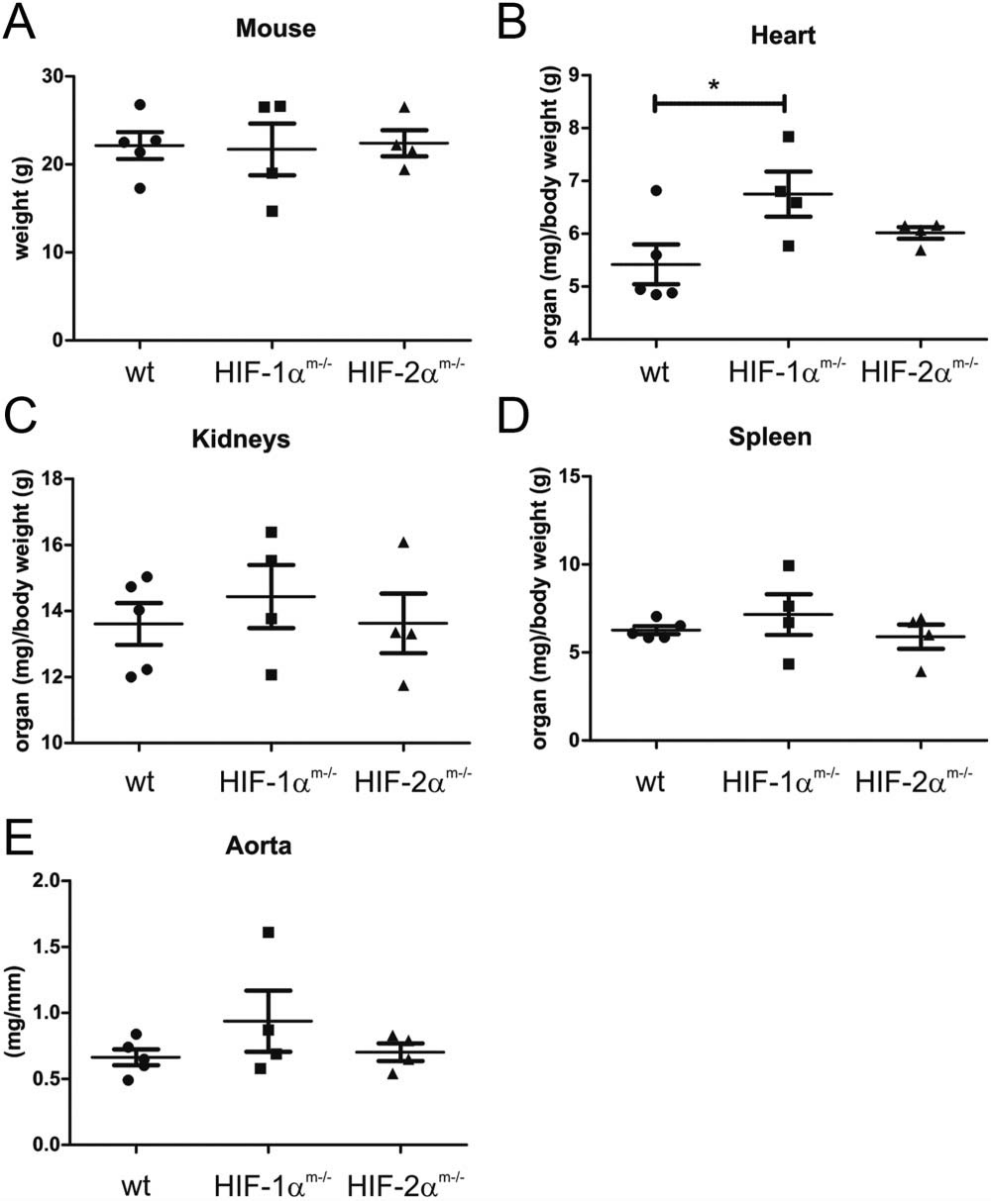
Angiotensin-induced atherosclerosis in ApoE^−/−^/myeloid Hif1α or Hif2α knockout mice after 7 days. Mice harboring an ApoE knockout (wt) combined with a myeloid Hif1α (Hif1α^m−/−^) or myeloid Hif2α (Hif2α^m−/−^) knockout were treated with angiotensin II (1.44 mg/kg/day) for 7 days and sacrificed to determine their body weight and the weight of their organs (A, B, C, D, E). The values are given as mean ± SEM from 4 to 5 individual animals. Significance was obtained from analysis of variance (ANOVA) and Bonferroni’s test. *P < 0.05.

Knockout of Hif-1α and -2α in ApoE^−/−^ mice was described earlier and confirmed by analysis of mRNA from spleen-derived macrophages (16, 17). When 3-month-old mice were infused with AngII at 1.44 mg/kg/day for 7 days, no differences were found in total body weight or the weight of kidneys or spleen (Fig. 1A, B, C, D). However, the expected hypertrophy in the left and right heart was more severe in Hif1α^m−/−^/ApoE^−/−^ mice when compared to the corresponding ApoE single knockout, leading to a significant increase in heart weight/body weight ratios, which was not observed in Hif2α^m−/−^/ApoE^−/−^ mice (Fig. 1B). This is accompanied by a tendency of increased aortic mass per length (mg/mm) (Fig. 1E) and may reflect a more severe aortic stiffness or calcification, as described earlier. In particular, HIF-1α affects the extracellular matrix, the scaffolding around cells, and the SMCs within the aorta, leading to increased stiffness (18).

**Figure 2:**
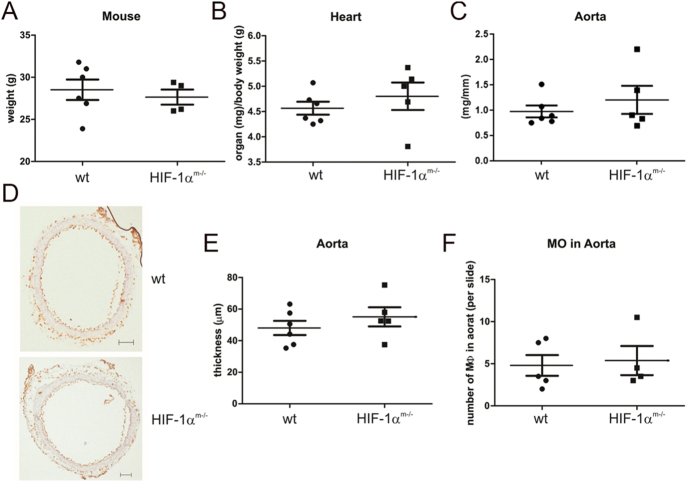
Angiotensin II-induced atherosclerosis in ApoE^−/−^/myeloid Hif1α knockout mice after 4 weeks. Mice harboring an ApoE knockout (wt: *n* = 6) or ApoE knockout combined with a myeloid Hif1α (Hif1α^m−/−^: *n* = 5) knockout were treated with angiotensin II (1.44 mg/kg/day) for 4 weeks and sacrificed to determine their body weight and the weight of their organs (A, B, C). Aorta were embedded, sectioned, and stained with F4/80 (brown) and Meyer’s hemalum (blue) (D). Aorta thickness (E) and the number of F4/80 positive cells (F) were determined from 10 slides and calculated as mean ± SEM per animal. The values are given as mean ± SEM from 4 to 5 individual animals.

Therefore, we decided to proceed with infusing Hif1α^m−/−^/ApoE^−/−^ and ApoE single knockout mice with 1.44 mg/kg/day AngII for 4 weeks. However, the differences observed before were lost with the prolonged treatment (Fig. 2A, B, C). Analysis of aortic wall hypertrophy and monocyte infiltration confirmed a non-significant, minor increase in aortic wall hypertrophy with no differences in the number of F4/80-positive cells (Fig. 2D, E, F). We concluded that the long-term response to AngII-induced hypertension was not dependent on myeloid Hif1α in the ApoE^−/−^ background. Our data indicate that Hif1α in macrophages is protective in the onset of AngII-induced vessel inflammation but not in established high-AngII states.

### HIF-1α but not HIF-2α is involved in plaque formation in ApoE^−/−^ mice

**Figure 3:**
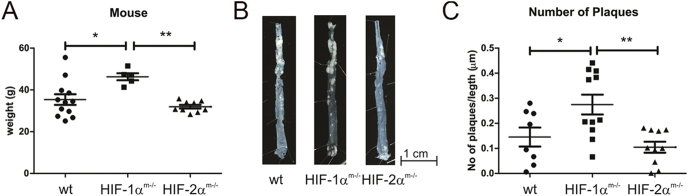
Twelve-month-old Hif1α^m−/−^/ApoE^−/−^ mice have higher plaque numbers. Animals without (ApoE^−/−^, wt) or with a myeloid Hif1α (ApoE^−/−^, Hif1α^m−/−^) or myeloid Hif2α (ApoE^−/−^, Hif2α^m−/−^) knockout fed a normal chow diet were sacrificed at 12 months of age and weighed (A). The aortae were removed, and the number of plaques was determined by manual counting at multiple anatomical sites, reflecting atherosclerotic onset and burden (B and C). The values are given as mean ± SEM for 11 individual animals per group. Significance was obtained from analysis of variance (ANOVA) with Bonferroni’s test. * = *P* < 0.05.

**Figure 4:**
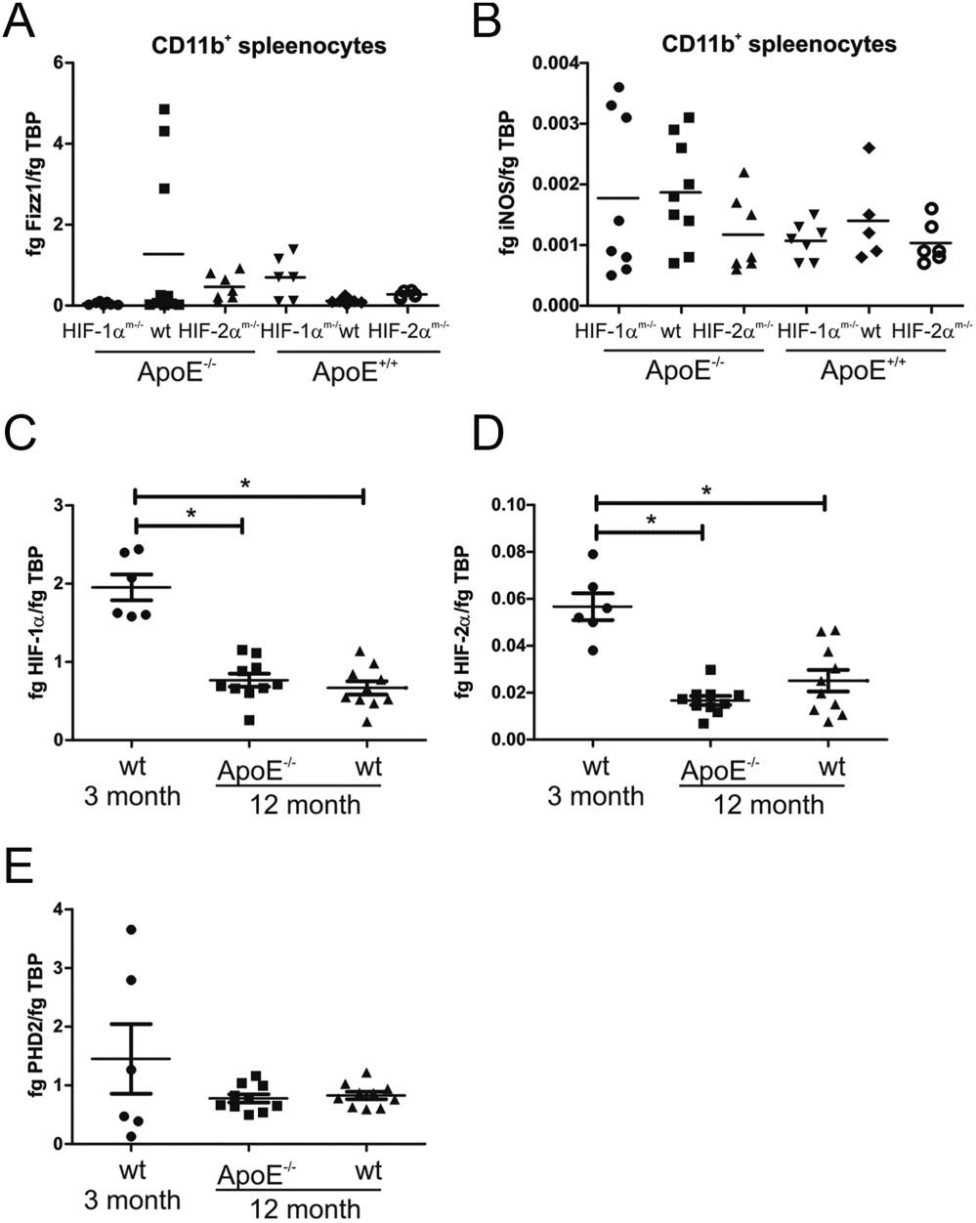
Twelve-month-old Hif1α^m−/−^/ApoE^−/−^ mice have a lower macrophage Hif1α and Hif2α expression but do not differ in iNOS or Fizz1 expression. CD11b^+^ cells were isolated from the spleen of mice harboring a myeloid Hif1α (Hif1α^m−/−^) or myeloid Hif2α (Hif2α^m−/−^) knockout combined with or without an ApoE knockout (ApoE^−/−^ or ApoE^+/+^) older than 12 months, or from 3-month-old mice with no knockout (wt), and mRNA was isolated and analyzed by qPCR for Fizz1 (A), iNOS (B), Hif1α (C), Hif2α (D), and PHD2 (E) expression. The values are normalized to TBP and given as mean ± SEM for 5–8 individual animals. Significance was obtained from analysis of variance (ANOVA) and Bonferroni’s test. * = *P* < 0.05.

In order to study the role of HIF in a more physiological and human relevant model of atherosclerosis, we analyzed HIF knockout mice in an ApoE^−/−^ background fed a normal chow diet for 12 months. Hif1α^m−/−^/ApoE^−/−^ double-knockout mice showed a significantly higher body weight when compared to ApoE single-knockout or Hif2α^m−/−^/ApoE^−/−^ mice (Fig. 3A). Importantly, Hif-1α^m−/−^/ApoE^−/−^ but not Hif2α^m−/−^/ApoE^−/−^ mice developed a significantly higher number of aortic plaques (Fig. 3B and C). Further analyses will be needed to clarify if the knockout of Hif1α in macrophages may influence the metabolic state of mice, which may influence body weight and sensitize the animals to the formation of atherosclerotic plaques. Plaque formation has been shown to be a consequence of macrophage activation and polarization, which in turn is supposed to be HIF-mediated (12, 16). Analysis of isolated CD11b^+^ cells from spleen from the 12-month-old animals, however, did not show any difference in the expression of iNOS, indicative of classical pro-inflammatory M1 polarization, or Fizz1, a classical marker for anti-inflammatory alternative M2 polarization (Fig. 4A and B). Major classical/alternative activation pathways were not altered in our model. Further investigation, including assessment of markers such as Arg1, CD206, and cytokine profiles, would be necessary to fully define macrophage phenotype. The interconnection of classical macrophage polarization and expression of metabolic genes (19) reinforces that polarization and metabolic function are intertwined.

Interestingly, the expression of Hif1α and Hif2α in macrophages was downregulated in old animals (3-month-old versus 12-month-old mice) irrespective of ApoE expression, while PHD2 level remained stable with age (Fig. 4D and E). This may indicate an impaired hypoxic response of macrophages in aged animals.

### Pseudotime analysis of human atherosclerotic macrophages supports context-dependent activation of HIF pathways

**Figure 5:**
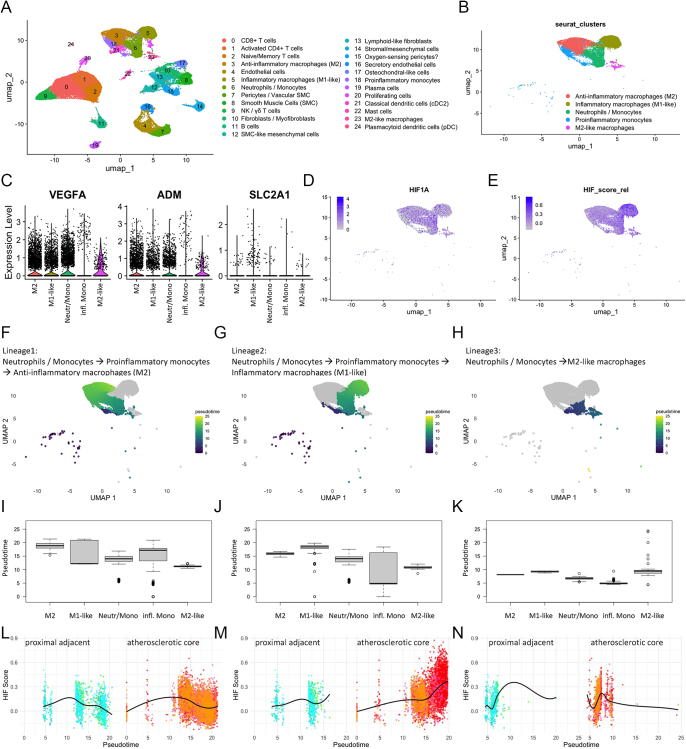
HIF activity dynamics in macrophages from normal and atherosclerotic human vessel wall regions. (A and B) UMAP embedding of (A) all cell clusters and (B) macrophage clusters from all patient samples. (C) Violin plots for expression of HIF target genes VEGFA, ADM, and SLC2A1 in identified macrophage clusters. (D and E) UMAPs colored by (D) HIF abundance and (E) HIF-score across the macrophage cluster HIF-score. (F, G, H) Inferred pseudotime using a root set in neutrophil-/monocyte-like cells progressing along three distinct trajectories and (I, J, K) lineage diagrams summarizing the three pseudotime trajectories for individual macrophage clusters. (L, M, N) Subset visualizations of HIF-score over pseudotime stratified by proximal adjacent (greenish) and atherosclerotic core (reddish) regions. Different color nuances depict different samples. (O, P, Q) Linage diagrams for HIF-score in the cell macrophage clusters differentially depicted for proximal adjacent (PA) and atherosclerotic core (AC).

To explore the transcriptional dynamics of hypoxia-responsive pathways in human normal and atherosclerotic vessel wall macrophages, we reanalyzed the publicly available single-cell RNA-sequencing dataset GSE159677 (15), comprising carotid endarterectomy specimens from three patients with matched proximal adjacent (PA) and atherosclerotic core (AC) regions. After unsupervised clustering and annotation of myeloid subpopulations (Fig. 5A), we extracted macrophage-related clusters (Fig. 5B) and performed pseudotime trajectory analysis. Using neutrophil-/monocyte-like cells as a root, three main differentiation lineages emerged: one toward inflammatory macrophages (M1-like), another toward anti-inflammatory macrophages (M2), and a third toward M2-like hybrid states (Fig. 5F–H). Overlaying a curated HIF target gene score (Fig. 5C–E) onto the pseudotime revealed that M2 cells appear in later pseudotime stages (Fig. 5F–K) and exhibited an increased expression of HIF-responsive genes in the atherosclerotic core region (Fig. 5L–N). Accordingly, within the plaque microenvironement, HIF signaling may be preferentially activated in macrophages acquiring anti-inflammatory features. These findings offer mechanistic support for the context-specific role of myeloid HIF-1α observed *in vivo* in mice in this study. In particular, the association of HIF activity with anti-inflammatory phenotypes aligns with the modest but consistent protective effects of HIF1α in advanced atherosclerosis, as seen in aged ApoE^−/−^ mice. These data collectively suggest that HIF signaling in macrophages may play a modulatory role during plaque progression, potentially favoring a reparative response under chronic inflammatory conditions.

## Discussion

The present study suggests that Hif1α in macrophages may protect against the development of aortic plaques in aged ApoE^−/−^ mice fed a chow diet. In AngII models, HIF-1α exhibits a context-dependent role: protective during early infusion but without significant impact during prolonged AngII exposure.

Two complementary perspectives have been postulated to explain the etiology of atherosclerosis. The ‘inside-out’ theory of atherosclerosis focuses on the intima as a primary site of damage and inflammation, while the ‘outside-in’ theory emphasizes the role of the adventitia and surrounding tissues (20). These perspectives are not mutually exclusive and together suggest that the role of HIF1 in atherosclerosis may be cell type- and model/context-specific.

The inside-out therory was previously described in the introduction to this article. Injury of the endothelial cell layer allow adhesion and invasion of proinflammatory cells at and into the vessel wall. Subsequent inflammation and lipid deposition within the vessel wall forces atherosclerosis (1, 2). The outside-in theory (21) emphasizes an impaired adventitial microcirculation as the main driver for atherosclerosis. Impaired microcirculation may be associated with lipid deposition in the vessel wall, attracting proinflammatory cells. In fact, tissue-resident macrophages may play a role in inflammation and adventitial microvessel disease. Although our data do not directly address adventitial microcirculation, the findings indicate that myeloid HIF responses are reduced in aged animals. Prior studies have shown that a decline in HIF activation correlates with impaired angiogenesis in various disease models (22). The enhancement of age-induced plaque formation by Hif1α knockout in the ApoE-deficient background shown here indicates a protective role of macrophage Hif1 in the aged vasculature. Mechanistically, an age-dependent increase in PHD3 expression was suggested as an underlying cause for reduced HIF1α protein accumulation (23). In contrast, macrophages show a downregulation of HIF1α and HIF2α mRNA without changes in PHD2 expression, which is the major HIF-regulating PHD isoform (24).

Both perspectives on atherosclerosis onset place a significant emphasis on the function of ECs. In a model of partial carotid ligation, the formation of atherosclerotic lesions and macrophage infiltration was reduced by endothelium-specific Hif1α^−/−^/ApoE^−/−^ knockout. From a mechanistic perspective, oxLDL instigates the endothelial expression of the chemokine, CXCL1 in an HIF-1α-dependent manner, which, in turn, prompts monocyte adhesion (25). Inhibition of PHDs, which facilitate the efficient degradation of HIFs, results in a consequential reduction in plaque formation (26). Indeed, FG4497, the pharmacologic agent employed to inhibit PHDs, demonstrates systemic activity, with the potential to engage target cells comprising endothelial and smooth muscle lineages, along with tissue-resident and circulating macrophages. In mice with a smooth muscle-specific knockout of HIF-1α, the remodeling and hypertrophy of SMCs induced by AngII was reduced. Furthermore, the knockout of Hif1α in SMCs has been shown to diminish AngII-induced fibrosis and macrophage infiltration (27). HIF-1α in SMCs protects the vessel wall from inflammation and atherosclerosis. Vascular inflammation and atherosclerosis in smooth muscle-specific ApoE/Hif1α double-knockout mice were reduced compared to WT/ApoE^−/−^ mice with a significantly lower infiltration of macrophages (28). Interestingly, Gong *et al.* conducted a meta-analysis of several published datasets and undertook lineage tracing studies in murine atherosclerosis models. The conclusion was reached that, in the context of atherosclerosis, there is potential for SMCs to undergo a process of differentiation into macrophages, which the authors attributed to an augmented NFκB activity (29). As NFκB alters HIF1α activity, it is intriguing to speculate that HIF-1α is essentially involved in SMCs for macrophage differentiation as well. 

A human histopathological study showed that hypoxia and HIF1α protein significantly co-localizes with macrophage-rich, inflammatory regions in advanced atherosclerotic plaques (30). These findings support the notion that HIF1α effects are context dependent, consistent with our observation that Hif1α is protective only in specific disease contexts (e.g. early AngII infusion onset, aged chow-fed mice). Aarup *et al.* demonstrated that myeloid-specific Hif1α expression in murine atherosclerotic lesions correlates with pro-inflammatory (M1) macrophage activation and promotes atherosclerosis. However, polarization toward the M2 macrophage phenotype was Hif1α independent. Our reanalysis of the GSE159677 dataset revealed that M2 polarized macrophages appear late in pseudotime with a slight increase in HIF activity in atherosclerotic lesions. Recent findings correlated M2 macrophages with plaque size, calcification, necrotic content, and a decrease in the number of vasa vasorum in the adventitia layer (31), indicating the ambivalent role of M2 macrophages in atherosclerosis. Actually HIF may not be involved in M2 polarization *per se*, but at least some protective M2-like features are HIF dependent, even if rather of a secondary nature, and alters inflammation. In fact, the weight gain and detrimental effect on atherosclerosis of Hif1α knock out in aged ApoE^−/−^ mice may reflect a chronic adaptation to metabolic stress, rather than classical anti-inflammatory polarization. We speculate, based on the prior literature (32), that HIF-1α may facilitate metabolic flexibility in macrophages under long-term lipid exposure.

In summary, our data provide evidence that macrophage Hif1α expression might be protective in the early onset of atherosclerosis, while with progression of disease, myeloid Hif1α has a minimal impact during prolonged AngII infusion. In contrast, development of atherosclerosis in aged mice on a chow diet is favored by reduced myeloid Hif1α expression. Our findings highlight important context dependence when reflecting prior work: HIF1α may have distinct roles at different disease stages and under different inflammatory loads. Future studies using transcriptomic or proteomic approaches in human specimens are needed to reconcile divergent findings across mouse models.

### Limitations and future directions

We did not assess the protein levels of HIF targets or plaque composition. Our interpretations are conservative and primarily descriptive of plaque burden and gross tissue effects. We did not assess plaque stability (e.g. necrotic core and fibrous cap), which limits mechanistic conclusions about vulnerability or remodeling. In addition, our conclusions are drawn cautiously and are suggestive rather than definitive regarding downstream signaling. Plaque analysis focused on manual counting of plaque number at multiple aortic sites as a measure of atherosclerotic onset and burden, which is reflected also in aortic mass per length (mg/mm). Histological characterization was limited to F4/80+ cell infiltration and did not include a detailed assessment of plaque composition, stability, or other inflammatory cell phenotypes. Consequently, potential effects of myeloid HIF signaling on plaque quality cannot be fully determined.

The genetic strategy employed targets myeloid cells using LysMCre-mediated recombination, which efficiently deletes Hif1α or Hif2α in macrophages and neutrophils but does not affect other plaque-resident cell types. In particular, vascular SMCs and SMC-derived macrophage-like cells, which have been reported to contribute substantially to plaque cellularity, are not targeted. As a consequence, residual HIF expression in non-myeloid cells may persist within the lesion and could attenuate the apparent impact of myeloid HIF deletion at the tissue level. This limitation would be expected to bias toward underestimating, rather than overestimating, myeloid-specific HIF effects.

Finally, the human single-cell RNA-Seq reanalysis was based on a limited number of patients and remains inherently associative. Although this analysis provides valuable translational context and supports macrophage heterogeneity and HIF-associated transcriptional programs in human plaques, it does not establish causality and should be interpreted accordingly.

Taken together, these limitations highlight that the present study primarily addresses the contribution of myeloid HIF signaling to overall plaque burden in specific experimental settings. Future studies combining broader genetic targeting strategies, deeper phenotypic characterization, and expanded human datasets will be required to fully elucidate cell type-specific and context-dependent roles of hypoxic signaling in atherosclerosis.

## Declaration of interest

The authors declare that there is no conflict of interest that could be perceived as prejudicing the impartiality of the work reported.

## Funding

The work was supported by grants from Deutsche Forschungsgemeinschafthttps://doi.org/10.13039/501100001659 (SFB 815 TP01 and TP08 and SFB 834).

## Author contribution statement

ND and KS designed the study, analyzed and interpreted the results, and prepared the manuscript.
